# Effect of the Emory Healthy Kitchen Collaborative on Employee Health Habits and Body Weight: A 12-Month Workplace Wellness Trial

**DOI:** 10.3390/nu16040517

**Published:** 2024-02-13

**Authors:** Sharon H. Bergquist, Danyang Wang, Rokhaya Fall, Jonathan P. Bonnet, Krystyna R. Morgan, Dominique Munroe, Miranda A. Moore

**Affiliations:** 1 Department of Medicine, Emory University, Atlanta, GA 30322, USA; shoresh@emory.edu (S.H.B.);; 2 Department of Family and Preventive Medicine, Emory University, Atlanta, GA 30322, USAjonathan.p.bonnet@emory.edu (J.P.B.); dominique.derek.munroe@emory.edu (D.M.); 3 Woodruff Health Sciences Center, Office of Well-Being, Emory University, Atlanta, GA 30322, USA; krystyna.rastorguieva@emoryhealthcare.org

**Keywords:** worksite wellness, teaching kitchen, health behaviors, nutrition, culinary medicine

## Abstract

Introduction: Teaching kitchens are being used to facilitate lifestyle changes with a focus on culinary and nutrition programs to improve health behaviors. Less is known regarding their use as a worksite wellness program and their influence on employees’ quality of life, body weight, and adoption of healthy behaviors. We evaluated changes in self-reported healthy behaviors, overall health, and weight during a one-year multidisciplinary teaching kitchen program. Methods: Thirty-eight benefits-eligible employees were recruited, screened based on a priori eligibility criteria that prioritized elevated body mass index (BMI), co-morbid conditions, and high levels of motivation to make lifestyle changes, and consented to participate in The Emory Healthy Kitchen Collaborative. This 12-month program included a 10-week didactic and experiential curriculum followed by continued support and access to health coaching implemented in an academic health system university hospital workplace between 2019 and 2020. Comparative statistics, paired *t*-test, Mcnemar’s tests, and Wilcoxon signed-rank tests were used to assess changes at four time points. Results: Participants improved diet quality (*p* ≤ 0.0001), increased confidence in tasting new foods (*p* = 0.03), and increased mindful eating habits (*p* = 0.00002). Significant changes were seen in physical activity levels; aerobic activities (*p* = 0.007), strength resistance activities (*p* = 0.02), and participation in yoga (*p* = 0.002). Most participants weighed within 5 lbs. of their starting weight at 3 months (*p* = 0.57). Conclusions: A teaching kitchen intervention is an innovative model for improving employee health behaviors and general health self-perception.

## 1. Introduction

Chronic diseases, such as heart disease, diabetes, and cancer, are the leading cause of disability and death in the United States (US), accounting for 90% of all deaths and 75% of healthcare costs in 2016 [1]. These costs are largely borne by employers, who provide insurance for over 50% of the US population [2]. Estimates suggest that the cost of unhealthy employees is USD 153 billion annually, with an additional USD 100 billion lost due to absenteeism and decreased productivity [3]. Improving employee health behaviors may be effective in reducing employers’ expenses, while simultaneously improving employee well-being, performance, and attendance. Given the significant public health implications and financial toll of chronic diseases, innovative tools have emerged to improve health outcomes in employees. Employer-sponsored worksite wellness programs have been widely adopted to improve employee health while mitigating health costs.

Previous studies on worksite wellness programs in diverse settings that contain incentives and incorporate interventions such as anthropometric screening, health coaching, and wellness classes on nutrition, physical activity, stress management, smoking cessation, alcohol consumption education, have been shown to lower body mass index (BMI), blood pressure, cholesterol, absenteeism, stress, and depression [4,5]. These findings suggest that worksite wellness programs are efficacious at reducing several risk factors associated with chronic diseases [5]. The outcome measures most frequently utilized by worksite wellness programs have included biomarkers such as lipid profiles, blood pressure, and body weight. Results using these measures have been mixed. However, these biomarkers often fail to measure changes in quality of life and behavioral health habits that may impact employee productivity and absenteeism. Assessments of changes in health behaviors, knowledge, attitudes, and self-efficacy are alternative relevant outcomes. These are underutilized indicators for evaluating program efficacy [4,5].

Interactive, team-based experiential teaching kitchen care delivery models may be particularly acceptable and effective in promoting sustained health behavior changes. Teaching kitchens are relatively novel, innovative, and engaging settings for building culinary skills, healthy eating habits, and self-efficacy, and may serve as focal points for skills training in other domains [6,7,8]. 

Teaching kitchens have been envisioned for use in various settings, including the workplace and hospitals [9]. Providing individuals with practical knowledge and skills in healthy cooking and nutrition and promoting healthy eating is predicted to improve overall health outcomes, reduce the risk of chronic diseases such as diabetes, obesity, and heart disease, and reduce healthcare costs. Recent studies on teaching kitchens have demonstrated favorable outcomes in increasing knowledge of healthy eating habits and improving dietary behaviors [7]. Patients with diabetes who completed a virtual culinary medicine curriculum exhibited significant improvements in their dietary habits, such as the increased consumption of fruits, vegetables, and whole grains, confidence in their ability to make healthy food choices and prepare meals at home, and decreased consumption of sugar-sweetened beverages and processed foods [10]. Public university students who enrolled in a semester-long nutrition course that used a teaching kitchen lab were found to have reduced stress levels and food insecurity [11].

Teaching kitchens utilized in workplace settings have also demonstrated efficacy in improving health behaviors. A 2019 study among Culinary Institute of America employees showed improvements in dietary habits, an increased consumption of fruits and vegetables, and confidence in cooking skills, following recipes, and tasting and preparing new foods [6]. Participants in a university employee wellness program experienced an increase in well-being, social connectedness, cooking self-efficacy, and comfort in the kitchen after program participation [12]. Teaching kitchens may also aid in improving BMI and related health outcomes. A virtual teaching kitchen program demonstrated reductions in BMI and improvements in metabolic health in individuals with diabetes [10].

The Emory Healthy Kitchen Collaborative (EHKC) was developed in 2019 as a 12-month multi-disciplinary, teaching-kitchen-based comprehensive lifestyle change program for promoting worksite wellness. Its rationale and design have previously been discussed [13]. Briefly, the EHKC is a robust self-care curriculum that includes didactic, experiential, and group learning sessions designed to comprehensively cover nutrition, culinary arts, exercise, yoga, mindful eating, stress resilience, and ethnobotany. Validated questionnaires were utilized to formally evaluate the impact of the program on commensurate health behaviors, along with traditional biomarkers. This allowed for a thorough analysis of the unique structure, content, and efficacy of this program on creating heath behavior change. Our primary aim was to evaluate Emory University and Emory Healthcare employee-reported changes in cooking confidence, dietary intake, mindful eating, physical activity, stress, and overall quality of life over 12 months. The secondary aim was to evaluate changes in weight and body composition [13].

## 2. Materials and Methods

### 2.1. Study Population

The participants recruited for this study were benefits-eligible Emory University and Emory Healthcare employees aged 18 to 65. To qualify for enrollment, applicants agreed to attend all program classes and study visits and consented to appear in videotapes and photographs taken during the teaching kitchen program. Applicants with any health condition that would significantly impact weight or limit participation (including bariatric surgery; pregnancy; inability to exercise due to a cardiovascular, pulmonary, orthopedic, or neurologic medical problem; or food allergies to gluten or nuts) were excluded. Among qualifying applicants (*n* = 184) who applied during a month-long recruitment period, 82 met the eligibility criteria. Grant funding and resources were limited, enabling the enrollment of only 41 applicants in the program. Consequently, based on a priori selection criteria that prioritized elevated BMI, co-morbid conditions, intent to utilize program resources and high motivation to make lifestyle changes (Supplemental Table S6), the participants were assigned a score; the 41 applicants with the highest scores were accepted into the program, with the remaining eligible candidates being placed on a wait list to serve as alternates (Supplemental Figure S2). 

### 2.2. Program Design and Facility

Emory faculty and staff who were subject matter experts in nutrition, culinary skills, physical activity, yoga, stress management, and mindful eating created a 20 h self-care curriculum [13]. The curriculum was delivered over 10 weeks at the beginning of the EHKC year-long program, which was deployed from 10 August 2019 to 10 August 2020. The curriculum consisted of five 4 h Saturday classes held every other weekend [14]. Each class included didactic and experiential sessions and concluded with a 30 min group session, during which participants ate a group-prepared, plant-based lunch using mindful eating techniques and engaged in discussions regarding the successes and challenges of implementing the skills taught during the program. Didactic instruction included three hours of nutrition education and one hour of instruction in the scientific rationale and practical application in each of the disciplines of yoga, physical activity, stress resilience, mindful-based eating, and ethnobotany. Experiential learning sessions included five hours of culinary skills training with hands-on cooking demonstrations, five half-hour mindful lunch sessions, a 1 h yoga session, and a 1 h group exercise session. Participants were encouraged to try various cooking techniques and self-care skills at home in between classes.

The sessions were held at the Emory University Hospital, where a “pop-up” kitchen was set up in the physician lounge. The kitchen consisted of eight individual cooking stations. An adjacent conference room was used for didactic sessions. The yoga and physical activity sessions were delivered in a yoga studio and veranda, both located in the hospital building.

For the remainder of the program year, participants had complimentary access to nightly yoga sessions on the Emory University campus, health promotion resources through Healthy Emory Connect (Emory’s digital health platform) [15], and virtual behavioral and health coaching through the Full Plate Living program (offered by the Ardmore Institute of Health) [16]. The health coaching consisted of optional group health coaching sessions (individual coaching was available at an additional cost to participants). Group support was offered through a private Facebook page and periodic events (such as a group potluck). Participants did not receive any financial incentives other than being entered in a draw for gift cards of USD 25 or USD 50 value (USD 300 in total) if they completed all program visits and survey assessments. The program was offered at no charge. All subjects gave their informed consent for inclusion before they participated in the study. The study was conducted in accordance with the Declaration of Helsinki, and the study protocol was approved by the Emory Institution Review Board (IRB 00109546 approved 4 December 2019) and registered with ClinicalTrials.gov (NCT04005495).

### 2.3. Assessment Instruments and Outcome Measures

Self-assessment survey responses and anthropometrics were obtained at baseline, 3 months, 6 months, and 12 months. The baseline (August 2019), 3-month (November 2019), and 6-month (February 2020) assessments took place on-site at the Paul W. Seavey Internal Medicine Clinic. As the 12-month assessment occurred in August 2021, during the COVID-19 pandemic lockdowns, several participants elected to not be assessed on-site; these participants were asked to self-report their anthropometric data. The self-assessment instruments included 6 survey questionnaires combined in 1 and deployed via Qualtrics (Provo, UT) [17]: Starting the Conversation (STC) [18], measuring dietary intake and cooking frequency and confidence [19]; Mindful Eating Questionnaire (MEQ) [20]; Physical Activity “Vital Sign” (PAVS) [21,22]; Perceived Stress Scale (PSS) [23]; and the MOS/RAND Health 36-Item Short Form [24], measuring global health, quality of life, and presenteeism. Scores and scale ranges are presented in Table 1. Anthropometrics included weight, body fat percentage, and visceral adipose tissue (VAT), which were measured with a non-invasive bioelectrical impedance device (Seca mBCA 514) [25]. The crosswalk between program activity and participant outcomes is presented as Table 2 in prior publications [13].

### 2.4. Statistical Analysis

Descriptive statistics (number and percentage of responses for categorical variables and mean, median, standard deviation [SD], and range for continuous variables) were calculated for all quantitative data collected (participant demographics, survey responses, and anthropometric data). Paired *t*-tests, Mcnemar’s tests, and Wilcoxon signed-rank tests were used based on the distribution of each measure and used to compare individual differences between study time points, with an alpha value of <0.05 considered statistically significant. All data analyses were performed using StataSE 14 (College Station, TX, USA) [26]. Power analysis was not performed, as this was a pilot without a control group. 

## 3. Results

Of the forty-one applicants accepted into the program, thirty-eight completed the study, resulting in a completion rate of 92.7%. Participant age ranged between 26 and 63 years old (mean, 48.0 years; SD, 10.0 years, Table 2). The majority were female (94.7%), African American (68.4%), followed by White (21.1%), and other (10.5%). All had at least a high school education. The mean BMI was 36.0 kg/m^2^ (SD 6.4). One participant mistakenly entered a higher weight into the eligibility survey; thus, one participant was weighted 20.6 kg/m^2^. Fewer than half (42.9%) had one or more co-morbidities. 

### 3.1. Behavioral Self-Assessments

**Figure 1:**
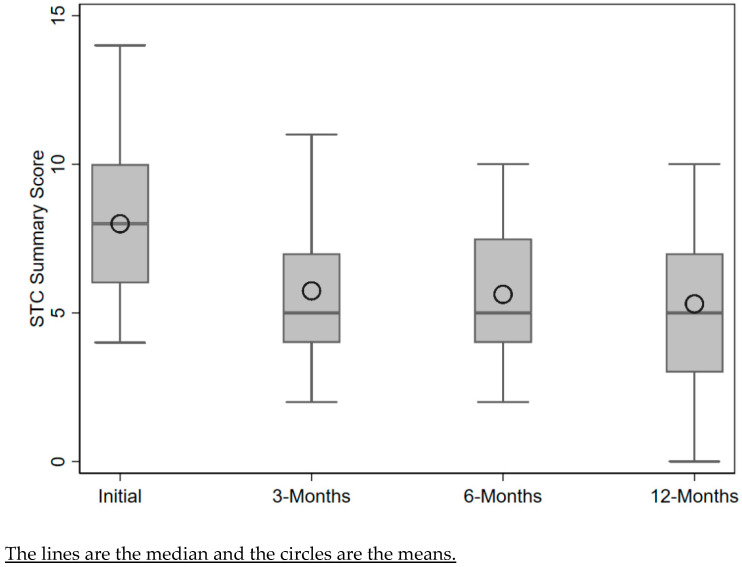
Change in Starting the Conversation scores from baseline to 12 months.

**Figure 2:**
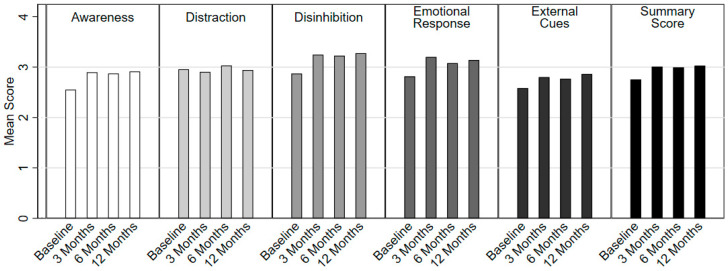
Changes in mindful eating score.

Diet quality improved throughout the program year, with the largest improvement in STC score (mean change −2.20, 95% confidence interval (CI) [CI] −3.00 to −1.40, *p* < 0.0001) from baseline to 3 months. The mean score continued to improve throughout the 12 months (mean change −2.74, 95% CI –3.94 to –1.54, *p* = 0.0001) (Figure 1 and Supplemental Table S1). Over the course of the 12-month trial, participants consumed fewer convenience and ready-made meals (28.95% to 13.0% and 52.63% to 13.04%, *p* = 0.06 and 0.01, respectively) and increased the frequency of preparing dishes from basic ingredients (68.42% to 82.61%, *p* = 0.25) (Table 3 and Supplemental Figure S1). The percentage of participants who prepared 4–6 meals per week from basic ingredients increased from 13.16% at the start of the program to 30.43% by the end. The number of participants who prepared meals from basic ingredients fewer than once a week decreased from 23.68% to 4.35%. A significant growth in participants’ confidence in tasting foods that they had not eaten before was seen at 6 months, as compared with the baseline (from 2.45 to 3.47, *p* = 0.03), and the confidence level remained higher than baseline at 12 months. Participants’ confidence in following a simple recipe increased from baseline to 6 months; however, these changes were not statistically significant (Figure 1 and Supplemental Table S2). Participants also showed significant increases in mindful eating habits, with mean scores increasing from a baseline of 2.76 to 3.01 (95% CI 0.13 to 0.37, *p* = 0.0002) at 3 months (Figure 2, Table 3 and Supplemental Table S2).

From baseline to 3 months, participants significantly increased aerobic activities (*p* = 0.007), strength resistance activities (*p* = 0.02), and participation in yoga (*p* = 0.002). However, when compared with the baseline, the 6- and 12-month physical activity levels showed no change (Supplemental Table S4).

The baseline mean PSS score of our participants was 13.13 (SD 7.63). Participants exhibited a decrease in stress perception following the 10-week curriculum; however, these levels increased over the subsequent months, with higher participant PSS scores at 12 months than at baseline (Supplemental Table S5). 

MOS/RAND Health 36-Item Short Form results from baseline to 3 months showed reductions in role limitation due to physical health, energy, emotional well-being, social functioning, and pain, as evidenced by higher scores (Supplemental Table S6). Additionally, role limitation scores due to emotional problems showed a slight increase from baseline to 6 months, indicating improvement. Additionally, the physical functioning and general health scores remained consistent from baseline to 12 months. However, these improvements in QOL patterns were not statistically significant. 

### 3.2. Anthropometric Assessments

From baseline to 3 months, 16% of participants decreased in weight by at least 5lbs. (mean change: −11.1; SD: 7.55), with 76% experiencing weight that stayed within a 5lbs. range up or down (mean change: 0.14; SD: 2.38; *p*-value: 0.57; Table 4). Due to the COVID-19 pandemic, in-person body composition analysis was only available for 14 participants at 12 months. From baseline to 3 months, 76% of participants experienced no change or an increase in their visceral adipose tissue (mean change: 0.57; SD: 0.64; *p*-value 0.06).

## 4. Discussion and Conclusions

This study demonstrated the efficacy of a multidisciplinary worksite teaching kitchen on employee health behaviors and general health perception. We saw significant improvements in healthy eating by study participants, which is in line with the effects of other teaching kitchen and nutrition education interventions. A 14-week nutrition course that included 50 min lectures and a hands-on teaching kitchen lab component in undergraduate students found a significant increase in fruit and vegetable intake among participants [11]. Another study in veterans, offering one- to twelve-session nutrition education courses with cooking demonstrations, showed positive improvements in fruit and vegetable consumption and the intake of beans and lentils. Our study results support previous findings showing the effectiveness of teaching kitchens with a focus on improving diet quality as a health behavior. The decrease in the consumption of convenience and ready-made meals and the increase in the frequency of preparing dishes from basic ingredients found in our study mirrors that found in other studies. For example, participants in a 16 h five-week employee wellness teaching kitchen program demonstrated an increase in preparing weekly meals from basic ingredients, cooking self-efficacy, and a decrease in the frequency of consuming fast-food meals and convenience foods [12]. A 14- to 16-week teaching kitchen program, with 2.5 weekly hours of instruction offered to Culinary Institute of America employees, found that their participants consumed fewer convenience and ready-made meals and showed an increase in the frequency of preparing dishes from basic ingredients [6]. Additionally, employees increased their confidence in following basic recipes and tasting new foods. These studies suggest that teaching kitchen interventions may be effective at promoting the adoption of healthier eating habits by reducing reliance on convenience and ready-made foods, and by increasing cooking self-efficacy and confidence in preparing meals from basic ingredients.

The significant increase in mindful eating found in our study participants is consistent with the findings from other behavioral interventions. An 8-week behavioral intervention program with a focus on mindful eating among overweight and obese premenopausal women showed an improvement in the awareness of hunger cues, decreased reliance on external cues for eating, and increased food enjoyment [27]. Moreover, a 6-week primary care mindful eating program found significant improvements in emotional eating, mindful eating, and a decrease in eating in response to external cues [28]. Our study findings suggest that teaching kitchens can similarly be effective in improving eating behaviors, including mindful eating.

Our participants’ physical activity outcomes may have been influenced by the COVID-19 pandemic. The inability to sustain increased physical activity throughout the culmination of the program is consistent with research showing the effect of lockdowns seen throughout the pandemic. Significant changes in physical [29,30] and sedentary activity [31] are a documented outcome of the lockdowns, and our results support the idea that COVID-19 had a significant impact on not only mental health, but also physical health. However, the increases in physical activity levels observed in EHKC participants in the first 3 months are similar to findings in an 8-week employee wellness program that included weekly educational sessions on nutrition and physical activity and pedometer-based walking activities measured from baseline, midpoint, and at the end of the implementation [32]. Moreover, an 8-week, 18-month-long worksite wellness program indicated an almost 50% increase in minutes of weekly physical activity in the intervention group when compared with the control group, which had a less than 15% improvement, similar to our 3-month findings [33]. Further studies using teaching kitchens are needed to explore whether the increase we observed initially would be sustained in the absence of lockdowns. 

Although not statistically significant, university employees who participated in a 12-week employee wellness program for obese and overweight participants, with follow-up after 26 weeks, demonstrated increases in quality-of-life qualifiers such as self-rated health, vitality days, and summative unhealthy days, similar to our findings [34]. Furthermore, the decrease in participants’ perceived stress levels from baseline to 3-month follow-up evidenced in our study aligns with the findings of a 10-week university-based teaching kitchen feasibility pilot study with a self-care curriculum and 30 h of instruction [6]. The increased level of stress at the 6 month follow-up and beyond could be influenced by the impact of stress associated with the COVID-19 pandemic (these visits took place in February 2020 and August 2020, overlapping with the first 6 months of the COVID-19 pandemic); the pandemic has been linked to elevated stress levels due to factors such as social isolation, financial insecurity, and fear of infection [35].

Despite a percentage of participants showing a decrease in BMI, the results were not statistically significant. Interestingly, while our results show a significant change in dietary and meal preparation habits, these were not consistently accompanied by weight loss. This may be partly because our curriculum was designed for teaching health behavior change rather than for achieving weight loss. Our results indicate that utilizing health behavior outcomes is valuable as an adjunct to anthropomorphic measures when assessing the efficacy of teaching kitchen programs.

### 4.1. Strengths and Limitations

The EHKC has several strengths that contribute to the existing teaching kitchen literature. First, the program was multi-disciplinary and offered a comprehensive and integrated approach to behavior change that differs from both traditional employee wellness programs and teaching kitchens solely focused on culinary skills. Secondly, the program was evidence-based, consisting of a 10-week intensive course and a 12-month follow-up. Scientific research was utilized to develop the design and content of the EHKC [13]. Thirdly, experiential learning was an important aspect of the program, complementing the didactic approach of typical worksite wellness programs, and increasing the likelihood for the adoption of new behaviors and increasing participants’ confidence [14]. Finally, the program focused on health behavior change rather than solely on traditional health marker metrics. This approach has been shown to promote long-term improvements in health issues rather than rely solely on biomarkers and medical symptom treatment.

This study is not without limitations. This study was conducted in a single workplace setting, which may limit the generalizability of the findings to other settings. The sample size was relatively small, which may have affected the statistical power of the study and limited the ability to detect significant differences between groups. As we performed multiple comparisons, our results which were statistically significant are to be taken as a signal of important associations. If we were to apply a multiple comparison correction, such as the Bonferroni correction (multiplying the *p*-value by the number of comparison tests ran on the data) [36], most of our statistically significant results would become non-significant.

Furthermore, the study relied on self-reported measures for dietary intake, physical activity, and stress levels, which may be subject to bias and inaccuracies. Additionally, participant recruitment prioritized motivated participants, which may have impacted the study outcomes and generalizability of findings. A lack of a control group limits our ability to make cause-and-effect claims and inferences on whether changes reported by participants are attributed to the EHKC or other external factors. Finally, a randomized controlled trial with a larger sample size and a longer follow-up period would provide more robust evidence of teaching kitchens’ effectiveness in promoting healthy behaviors and improving health outcomes.

Further research is needed to investigate the impact of teaching kitchens with a larger sample size and longer duration to assess the sustained program impact over time. This would allow researchers to determine whether reported behavior changes in participants are maintained over time and with diverse populations. Importantly, it would be valuable to see how impactful these sustained health behavior changes are on health outcomes over time. There is a need for further research on effective stress management interventions to sustain stress reduction in the long term, especially in the context of the ongoing COVID-19 pandemic. Lastly, targeted programming aimed at specific metrics may also be more beneficial in seeing substantial improvements.

### 4.2. Conclusions

Teaching kitchens are a novel way to influence health behaviors when delivered as a worksite wellness program to employees. The EHKC 12-month pilot multi-behavior change teaching kitchen program delivered to employees significantly improved self-reported cooking confidence, dietary patterns, and physical activity in participants. Although perceived stress levels were initially improved, the reduction was not sustained, possibly influenced by the COVID-19 pandemic. Our findings indicate that teaching kitchens are a viable and enjoyable means by which employers can increase their employees’ self-assessed general health.

## Figures and Tables

**Table 1:** Self-assessment instruments for measuring behavior change and health perception.

Survey Instrument	Domain Assessed	Survey Description
Starting The Conversation (STC)	Dietary intake	Eight-item simplified food frequency instrument designed for use in primary care and health-promotion settings assessing dietary patterns with regard to the intake of (1) fast food (2) fruit (3) vegetables (4) soda/sweet tea (5) beans, chicken, fish (6) snack chips, crackers (7) desserts, and (8) margarine, butter, and meat fat. Scores range from 0 to 16, with lower scores indicating a higher intake of healthy foods and lower intake of dietary components with adverse effects on health.
A cooking skills questionnaire developed by Barton et al. (2011)	Cooking frequency and confidence	Eight questions from this seventeen-item questionnaire were used to assess cooking frequency, confidence in preparing new recipes from basic ingredients, and confidence in reading nutrition labels.
Mindful Eating Questionnaire (MEQ)	Mindful eating	Twenty-eight-item questionnaire evaluating disinhibition, awareness, external cues, emotional response, and distraction during meal consumption. Higher scores indicate greater mindful eating practice.
Physical Activity “Vital Sign” (PAVS)	Physical activity	Two questions, adapted from the Behavioral Risk Factor Surveillance System (BRFSS) and validated to screen for inactivity in clinical settings, with an optional third question assessing strength resistance exercise. We added three questions assessing the frequency and duration of yoga practice and plans to be more physically active in the next six months.
Perceived Stress Scale (PSS)	Perceived stress	Ten-item psychological instrument assessing an individual’s perception of stress over the past month and appraisal of the degree to which life situations are stressful. Scores on the PSS can range from 0 to 40, with higher scores indicating greater perceived stress.
MOS/RAND Health 36-Item Short Form	Global health, quality of life, and presenteeism	Items cover eight health areas: physical functioning, bodily pain, role limitations due to physical health problems, role limitations due to personal or emotional problems, emotional well-being, social functioning, energy/fatigue, and general health perceptions. Scores range from 0 and 100, with higher scores representing a more favorable state of health.

**Table 2:** Demographic characteristics of participants.

Characteristic	Participants
	n (%)
Age (years), mean (SD), median {range}	48.0 (10.0), 49.5 {26–63}
≤40	7 (18.4)
41–50	13 (34.2)
51–60	14 (36.8)
>60	4 (10.5)
Gender	
Female	36 (94.7)
Male	2 (5.3)
Race	
White	8 (21.1)
African American	26 (68.4)
Other	4 (10.5)
Marital status	
Single, never married	15 (39.5)
Married or domestic partnership	13 (34.2)
Other	10 (26.3)
Highest level of education	
High school graduate	0 (0.0)
Some college credit, no degree	10 (26.3)
Bachelor’s degree	14 (36.8)
Trade/technical vocational training	4 (10.5)
Masters/doctoral degree	10 (26.3)
Total household income	
USD 10,000 to USD 24,999	0 (0.0)
USD 25,000 to USD 49,999	10 (26.3)
USD 50,000 to USD 74,999	10 (26.3)
USD 75,000 to USD 99,999	9 (23.7)
USD 100,000 and greater	7 (18.4)
Unknown	2 (5.3)
Number of people in the household	
1	7 (18.4)
2	14 (36.8)
3	8 (21.1)
4 or more	9 (23.7)
Children aged under 16 years in the household	
0	25 (65.8)
1	9 (23.7)
2 or more	4 (10.5)
BMI, mean (SD), median {range}	36.0 kg/m^2^ (6.4), 36.4 kg/m^2^ {20.6–50.3}
Healthy	1 (2.63)
Overweight	5 (13.16)
Obese	32 (84.21)
Comorbidities	
Diabetes	6 (15.8)
Hypertension	21 (55.3)
Hyperlipidemia	11 (29)
Cancer	6 (15.8)
Heart disease	2 (5.3)
Number of comorbidities	
None	20 (57.1)
1	9 (25.7)
2 or more	6 (17.2)

BMI, body mass index; SD, standard deviation.

**Table 3:** Changes in cooking frequency and confidence.

	Initial (n = 38)	3 Months (N = 35)	6 Months (N = 32)	12 Months (N = 23)	Initial to 3 Months Change *p*-Value	Initial to 6 Months Change *p*-Value	Initial to 12 Months Change *p*-Value
Frequency questions, N (%)							
What kind of cooking do you do at the moment?							
Cook convenience foods and ready meals	11 (28.95)	7 (20.00)	4 (12.50)	3 (13.04)	0.16	0.10	0.06
Put together ready-made ingredients to make a complete meal (e.g., use ready-made sauces)	20 (52.63)	13 (37.14)	12 (37.50)	3 (13.04)	0.20	0.17	0.01
Prepare dishes from basic ingredients	26 (68.42)	25 (71.43)	22 (68.75)	19 (82.61)	0.71	0.78	0.25
Other, please specify	1 (2.63)	1 (2.86)	0 (0.00)	0 (0.00)	-	-	-
Do not cook at all	0 (0.00)	0 (0.00)	1 (3.13)	0 (0.00)	-	-	-
In a normal week, how often do you prepare and cook a main meal from basic ingredients, for example, making Shepherd’s Pie starting with raw mince and potatoes?							
Daily	1 (2.63)	1 (2.86)	1 (3.13)	4 (17.39)	0.02	0.17	0.007
4–6 times a week	5 (13.16)	8 (22.86)	7 (21.88)	7 (30.43)			
2–3 times a week	11 (28.95)	13 (37.14)	13 (40.63)	8 (34.78)			
Once a week	10 (26.32)	11 (31.43)	9 (28.13%)	3 (13.04)			
Less than once a week	9 (23.68)	2 (5.71)	2 (6.25)	1 (4.35)			
Never	2 (5.26)	0 (0.00)	0 (0.00)	0 (0.00)			
Confidence Questions, mean (SD)							
How confident do you feel about being able to cook from basic ingredients?	3.00 (3.07)	2.43 (2.95)	2.88 (3.54)	2.48 (3.38)	0.28	0.89	0.80
How confident do you feel about following a simple recipe?	2.05 (2.76)	2.37 (3.17)	2.94 (3.80)	2.17 (3.38)	0.83	0.15	0.67
How confident do you feel about tasting foods that you have not eaten before?	2.45 (2.41)	3.00 (2.57)	3.47 (3.23)	3.22 (3.37)	0.50	0.03	0.12
How confident do you feel about preparing and cooking new foods and recipes?	3.08 (2.95)	2.69 (2.79)	3.00 (3.18)	2.96 (3.13)	0.43	0.96	0.96

**Table 4:** Changes in body composition.

	Initial to 3 Months				Initial to 6 Months				Initial to 12 Months			
	*n*	Mean Change (SD)	Median {Range}	*p*	*n*	Mean Change (SD)	Median {Range}	*p*	*n*	Mean Change (SD)	Median {Range}	*p*
Weight												
≥5lbs loss	6	−11.10 (7.55)	−7.78 {−25.69, −5.4}	0.57	6	−16.26 (18.96)	−7.72 {−54.24, −5.62}	0.72	4	−26.07 (30.89)	−12.18 {−72.21, −7.72}	0.99
<5lbs loss/gain	28	0.14 (2.38)	−0.06 {−4.52, 4.52}		15	−0.39 (1.82)	−0.99 {−3.86, 2.31}		5	0.68 (3.36)	2.97 {−3.53, 3.2}	
≥5lbs gain	3	8.96 (2.55)	8.28 {6.83, 11.79}		11	11.51 (8.63)	7.49 {5.73, 33.62}		5	20.46 (21.18)	12.68 {6.61, 57.98}	
Relative Fat Mass Value												
Decreased	16	−1.13 (0.88)	−0.88 {−3.24, −0.21}	0.25	12	−1.55 (1.75)	−0.74 {−6.01, −0.04}	0.41	5	−2.72 (3.54)	−1.27 {−8.92, −0.45}	0.78
No change/ Increased	21	1.39 (0.96)	1.33 {0.19, 3.12}		20	1.39 (1.18)	1.075 {0.04, 4.59}		9	1.91 (2.03)	1.54 {0.06, 6.94}	
Skeletal Muscle Mass Value												
Decreased	22	−1.98 (1.44)	−1.39 {−6.43, −0.30}	0.12	17	−1.89 (2.10)	−1.42 {−9.15, −0.27}	0.68	7	−3.78 (4.39)	−1.72 {−13.5, −0.7}	0.95
No change/ Increased	15	1.49 (1.22)	1.66 {0.08, 4.34}		15	1.72 (1.86)	1.12 {0, 6.85}		7	3.95 (3.05)	3.85 {1.1, 9.09}	
Visceral Adipose Tissue Value												
Decreased	9	−0.67 (0.80)	−0.50 {−2.49, −0.03}	0.06	15	−0.59 (0.55)	−0.35 {−1.97, −0.09}	0.68	6	−1.02 (0.96)	−0.74 {−2.53, −0.07}	0.92
No change/ Increased	28	0.57 (0.64)	0.30 {0.04, 2.49}		17	0.63 (0.52)	0.39 {0.06, 1.66}		8	0.83 (1.29)	0.35 {0.09, 3.94}	

## Data Availability

The article contents have not been previously presented. Data are available from the authors upon reasonable request.

## Supplementary Materials

The following supporting information can be downloaded at: https://www.mdpi.com/article/10.3390/nu16040517/s1, Figure S1: Changes in cooking frequency and confidence; Figure S2: Emory Healthy Kitchen Collaborative (EHKC) Recruitment Overview; Table S1: Changes in the Starting the Conversation (STC) questionnaire scores; Table S2: Changes in mindful eating from baseline to 12 months; Table S3: Changes in physical activity from baseline to 12 months; Table S4: Changes in perceived stress from baseline to 12 months; Table S5: Changes in global health, quality of life, and presenteeism from baseline to 12 months; Table S6: Participant Interest Survey/Ranking Criteria.
