# Supplementary material for: Effect of the Emory Healthy Kitchen Collaborative on Employee Health Habits and Body Weight: A 12-Month Workplace Wellness Trial
Source: Nutrients. 2024 Feb 13;16(4):517. doi: 10.3390/nu16040517 (PMC10892851; doi:10.3390/nu16040517)

SUPPLEMENTAL TABLES AND FIGURES

Supplemental Table S1. Changes in the Starting the Conversation (STC) questionnaire scores

Summary Score Change	Initial to 3 months (n = 35)	Initial to 6 months (n = 32)	Initial to 12 months (n = 23)
Mean change	-2.20	-2.25	-2.74
SD	2.32	2.86	2.78
95% CI	(-3.00, -1.40)	(-3.28, -1.22)	(-3.94, -1.54)
p-value	<0.0001	0.0001	0.0001

Note. SD = standard deviation; CI = confidence interval.

Supplemental Table S2. Changes in mindful eating from baseline to 12 months

	Initial<br>N=38	3 Months<br>N=35	6 Months<br>N=32	12 Months<br>N=23
**Awareness**				
Mean (SD)	2.55 (0.57)	2.90 (0.46)	2.87 (0.52)	2.91 (0.53)
Median [range]	2.43 [1.29-3.86]	3.00 [2.00-3.71]	2.86 [1.57-4.00]	3.00 [1.71-4.00]
**Distraction**				
Mean (SD)	2.96 (0.63)	2.91 (0.52)	3.03 (0.55)	2.94 (0.70)
Median [range]	3.00 [1.33-4.00]	3.00 [1.33-3.67]	3.00 [1.33-4.00]	3.00 [1.67-4.00]
**Disinhibition**				
Mean (SD)	2.87 (0.56)	3.25 (0.47)	3.23 (0.44)	3.28 (0.44)
Median [range]	2.81 [1.75-4.00]	3.25 [2.38-4.00]	3.13 [2.50-4.00]	3.25 [2.38-4.00]
**Emotional**				
Mean	2.82 (0.77)	3.20 (0.54)	3.08 (0.69)	3.14 (0.52)
Median [range]	3.00 [1.25-4.00]	3.25 [1.75-3.75]	3.00 [1.75-4.00]	3.00 [2.00-4.00]
**External**				
Mean (SD)	2.58 (0.61)	2.80 (0.60)	2.77 (0.70)	2.86 (0.77)
Median [range]	2.50 [1.50-4.00]	2.67 [1.67-4.00]	2.67 [1.50-4.00]	2.83 [1.33-4.00]
**Summary score**				
Mean (SD)	2.76 (0.37)	3.01 (0.32)	3.00 (0.31)	3.03 (0.36)
Median [range]	2.78 [1.79-3.63]	3.04 [2.28-3.50]	3.05 [2.32-3.65]	3.05 [2.33-3.76]
**Summary score change**				
Mean (SD)	Reference	0.25 (0.35)	0.23 (0.30)	0.27 (0.29)
95% CI	Reference	(0.13, 0.37)	(0.12, 0.34)	(0.15, 0.40)
p	Reference	0.0002	0.0002	0.0002

Supplemental Table S3. Changes in physical activity from baseline to 12 months

Question	Initial		3 Months		6 Months		12 Months		Initial to 3 months change *p*-value	Initial to 6 months change *p*-value	Initial to 12 months change *p*-value
	N	%	N	%	N	%	N	%			
On average, how many days per week do you engage in moderate to strenuous exercise											
0 days	6	15.79	2	5.71	6	18.75	4	18.18	0.007	0.99	0.99
1 - 2 days	15	39.47	9	25.71	11	34.38	5	22.73			
3 - 4 days	10	26.32	13	37.14	10	31.25	7	31.82			
5+ days	7	18.42	11	31.43	5	15.63	6	27.27			
On average, how many minutes do you engage in exercise at this level?									0.55	0.48	0.39
0min	6	15.79	3	8.57	5	15.63	3	15.00			
1-29min	9	23.68	11	31.43	5	15.63	3	15.00			
30-44min	13	34.21	9	25.71	10	31.25	6	30.00			
45+min	10	26.32	12	34.29	12	37.50	8	40.00			
How many days a week do you perform muscle strengthening exercises, such as bodyweight exercises or resistance training?											
0 days	29	78.95	16	45.71	20	62.50	14	63.64	0.02	0.42	0.51
1 - 2 days	6	15.79	13	37.14	7	21.88	4	18.18			
3 - 4 days	2	5.26	5	14.29	5	15.63	3	13.64			
5+ days	0	0	1	2.86	0	0.00	1	4.55			
How many days /months do											

you participate in a yoga class?											
Yes (>=1)	3	7.89	12	34.29	6	18.75	5	22.73	0.002	0.25	0.13
No (0)	35	92.11	23	65.71	26	81.25	17	77.27			
On average, how many minutes do you engage in yoga during each class?											
0min	32	88.89	20	58.82	24	77.42	15	71.43	0.001	0.25	0.38
1-29min	1	2.78	2	5.88	0	0.00	1	4.76			
30-44min	0	0.00	3	8.82	1	3.23	0	0.00			
45+min	3	8.33	9	26.47	6	19.35	5	23.81			
Do you plan to be more physically active in the next 6 months?											
Yes	38	100.00	35	100.00	31	96.88	22	95.65	-	-	-
No	0	0.00	0	0.00	1	3.13	1	4.35			

Supplemental Table S4. Changes in perceived stress from baseline to 12 months

	Initial (N=38)	3 Months (N=35)	6 Months (N=32)	12 Months (N=23)
**PSS score**				
Mean (SD)	13.13 (7.63)	12 (5.86)	12.84 (7.05)	13.96 (8.03)
Median [range]	14 [0-32]	12 [0-24]	13 [0-24]	16 [0-26]
**PSS score change**				
Mean change (SD)	Reference	-0.89 (5.96)	0.22 (6.72)	0.09 (7.97)
95% CI	Reference	(-2.93, 1.16)	(-2.20, 2.64)	(-3.36, 3.53)
p	Reference	0.39	0.86	0.96

Supplemental Table S5. Changes in global health, quality of life, and presenteeism from baseline to 12 months

Summary Score	Initial (N = 38)	3 Months (N = 35)	6 Months (N = 32)	12 Months (N = 23)
**Physical functioning**				
Mean (SD)	81.84 (15.74)	85.14 (21.13)	82.81 (19.55)	83.26 (18.00)
Median [range]	85 [40-100]	95 [25-100]	90 [30-100]	90 [45-100]
**Role limitations due to physical health**				
Mean (SD)	82.24 (28.42)	87.86 (25.27)	82.03 (29.94)	77.17 (38.37)
Median [range]	100 [0-100]	100 [0-100]	100 [0-100]	100 [0-100]
**Role limitations due to emotional problems**				
Mean (SD)	80.7 (34.34)	76.19 (37.55)	81.25 (30.45)	79.71 (32.94)
Median [range]	100 [0-100]	100 [0-100]	100 [0-100]	100 [0-100]
**Energy/ fatigue**				
Mean (SD)	56.05 (18.53)	58.43 (18.46)	57.19 (19.75)	54.13 (23.04)
Median [range]	60 [20-85]	65 [5-85]	57.5 [10-85]	50 [15-90]
**Emotional well being**				
Mean (SD)	77.58 (14.21)	79.66 (13.46)	77.13 (17.19)	74.78 (18.66)
Median [range]	80 [48-96]	84 [44-100]	82 [36-100]	80 [40-100]
**Social functioning**				
Mean (SD)	77.63 (24.69)	83.57 (20.95)	83.20 (18.41)	73.37 (29.02)
Median [range]	87.5 [12.5-100]	100 [25-100]	87.5 [50-100]	75 [0-100]
**Pain**				
Mean (SD)	74.21 (19.61)	74.93 (22.35)	73.98 (21.14)	68.80 (31.65)
Median [range]	72.5 [35-100]	80 [22.5-100]	73.75 [32.5-100]	80 [0-100]
**General Health**				
Mean (SD)	61.78 (19.09)	67.14 (17.42)	67.19 (18.88)	66.52 (16.82)
Median [range]	65 [20-95]	70 [25-95]	72.5 [25-95]	70 [25-90]
**Overall score**				
Mean (SD)	74.00 (14.82)	76.61 (14.96)	75.60 (14.89)	72.22 (19.67)
Median [range]	76.42 [42.48-94]	82.75 [37.19-94.75]	78.64 [36.69-95.25]	75.13 [32.33-95.63]
**Overall score change**				
Mean (SD)	Reference	2.31 (2.32)	2.07 (2.28)	0.72 (3.36)
95% CI	Reference	(-2.40, 7.03)	(-2.58, 6.71)	(-6.23, 7.68)
p	Reference	0.33	0.37	0.83

Supplemental Table S6. Participant Interest Survey/ Ranking Criteria

Which of the following describes you best:
I want to improve my lifestyle (healthier diet and/or improve physical activity), but don't know when I will
I really want to improve my lifestyle (healthier diet and/or improve physical activity) and I want to start now
Do you have diabetes?
Do you have any personal history of heart disease?
Do you have high cholesterol?
Do you have hypertension?
Are you prescribed a medication for blood pressure?
Do you have a history of cancer?
On a scale of 1-10, with 1 being the lowest and 10 being the highest, how would you rate your motivation to change towards a healthier lifestyle?
On a scale of 1-10, with 1 being the lowest and 10 being the highest, how would you rate your motivation to make more home prepared meals each week?
On a scale of 1-10, with 1 being the lowest and 10 being the highest, how would you rate your motivation to make more home prepared meals each week?
On a scale of 1-10, with 1 being the lowest and 10 being the highest, how would you rate your motivation to attend free yoga classes offered virtually through Yoga at EUH program?
On a scale of 1-10, with 1 being the lowest and 10 being the highest, how would you rate your motivation to work with an online health coach each week?
How did you hear about the program? (*Please be as specific as you can.)

Supplemental Figure S1. Changes in cooking frequency and confidence

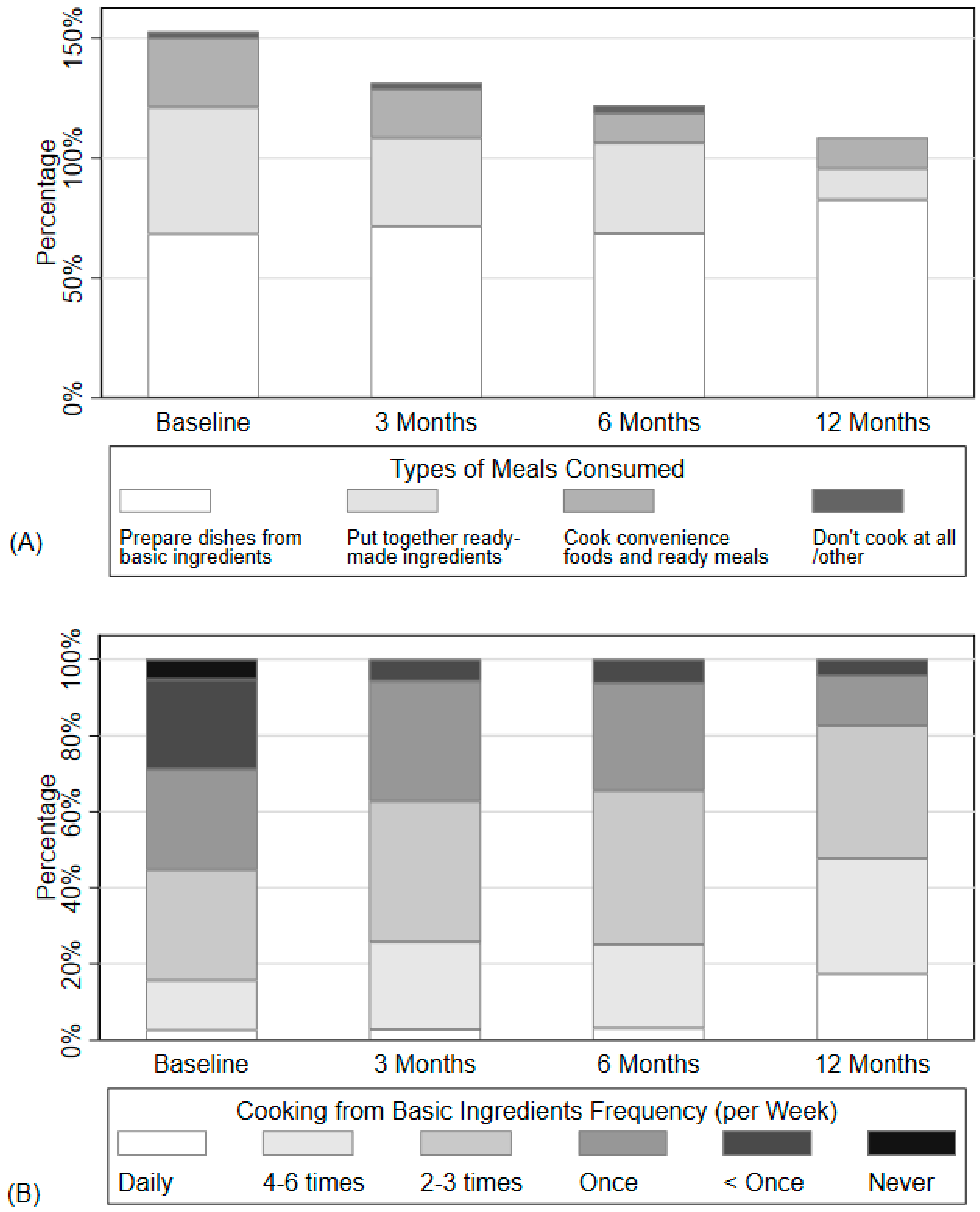

Supplemental Figure S2. Emory Healthy Kitchen Collaborative (EHKC) Recruitment Overview

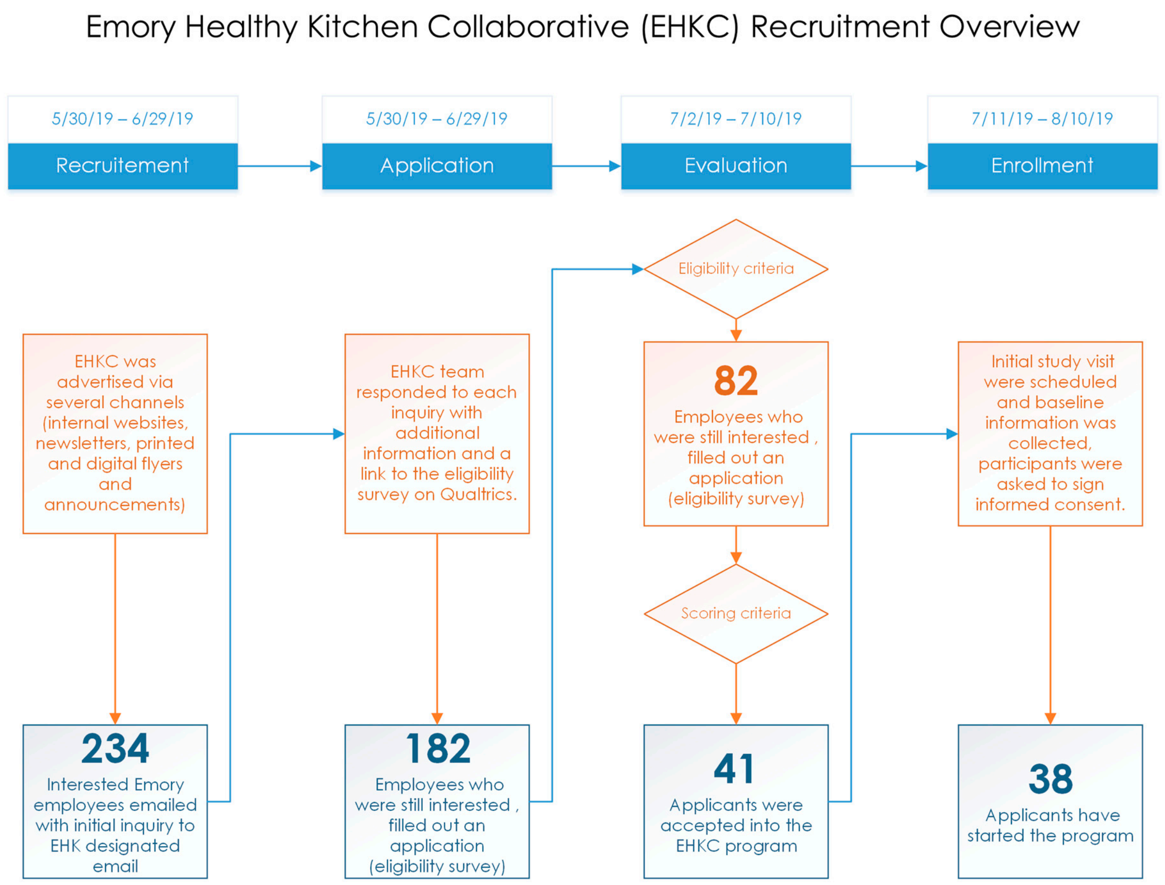

Supplement: Supplementary file 1 [file nutrients-16-00517-s001.zip › nutrients-2827436-supplementary.pdf]
